# Integrating Nutritional Psychiatry into Mental Health Practice: Insights from Italian Mental Health Professionals

**DOI:** 10.1192/j.eurpsy.2026.11677

**Published:** 2026-07-31

**Authors:** A. Litta, A. Nannavecchia, A. Vacca, M. V. Mino’, V. Favia, A. Ventriglio

**Affiliations:** 1 Department of Precision and Regenerative Medicine and Ionian Area (DiMePRe-J), University of Bari “Aldo Moro”, Bari; 2 Mental Health Department, Asl Taranto, Taranto; 3 AReSS Puglia- Regional Strategic Agency for Health and Social, Bari; 4 sychiatric Rehabilitation Center “Don Tonino Bello”- Assoc. M.I.T.A.G. Onlus, Brindisi; 5 Istituto Tumori Giovanni Paolo II, IRCCS, National Cancer Institute, Bari; 6 Department of Clinical and Experimental Medicine, University of Foggia, Foggia, Italy

## Abstract

**Introduction:**

Nutritional psychiatry emphasizes the role of diet in modulating brain function, emotional regulation, and psychiatric outcomes. Despite increasing international evidence, the integration of nutrition into psychiatric care remains limited, partly due to insufficient training among mental health professionals.

**Objectives:**

This study aimed to explore how Italian psychiatrists and psychologists perceive the role of nutritional psychiatry in their clinical practice, and to investigate which strategies they consider most relevant for implementing dietary interventions within mental health care.

**Methods:**

An adapted Italian version of the questionnaire developed by Mörkl et al. (2021) was administered online to a voluntary sample of 110 mental health professionals. The survey included items on nutritional knowledge, attitudes toward nutrition in psychiatry, and implementation of dietary and lifestyle interventions. Data were analyzed using non-parametric statistical tests, with significance set at p < 0.05.

**Results:**

Participants rated the nutritional status of patients with psychiatric disorders as “low” or “very low,” with no significant differences between psychiatrists and psychologists. Metabolic screening programs were reported by only 20% of respondents, despite 83% considering them highly useful. Nutritional interventions were occasionally used by 57% of professionals and regularly by 18%, with psychiatrists reporting significantly higher engagement than psychologists (p < 0.05). Recommendations most often included Mediterranean diets (31%), lifestyle counselling, and dietary supplements, though these practices were rarely standardized. Female psychiatrists reported significantly higher self-rated knowledge of nutritional psychiatry compared to other groups (p < 0.05).

**Conclusions:**

Italian mental health professionals acknowledge the importance of nutrition in psychiatric care but report limited training and inconsistent practices. Bridging this gap may benefit from structured education, stronger interdisciplinary collaboration, and the formal inclusion of dietitians within mental health teams. The development of evidence-based guidelines and the implementation of metabolic screening protocols could further strengthen the integration of nutritional psychiatry into everyday clinical practice

**Disclosure of Interest:**

None Declared

